# Identification of Natural Inhibitors Against SARS-CoV-2 Drugable Targets Using Molecular Docking, Molecular Dynamics Simulation, and MM-PBSA Approach

**DOI:** 10.3389/fcimb.2021.730288

**Published:** 2021-08-12

**Authors:** Prem Prakash Kushwaha, Atul Kumar Singh, Tanya Bansal, Akansha Yadav, Kumari Sunita Prajapati, Mohd Shuaib, Shashank Kumar

**Affiliations:** Molecular Signaling & Drug Discovery Laboratory, Department of Biochemistry, Central University of Punjab, Bathinda, India

**Keywords:** isoquercetin, 10-hydroxyaloin A, SARS-CoV-2, *in silico*, drugable targets

## Abstract

The present study explores the SARS-CoV-2 drugable target inhibition efficacy of phytochemicals from Indian medicinal plants using molecular docking, molecular dynamics (MD) simulation, and MM-PBSA analysis. A total of 130 phytochemicals were screened against SARS-CoV-2 Spike (S)-protein, RNA-dependent RNA polymerase (RdRp), and Main protease (M^pro^). Result of molecular docking showed that Isoquercetin potentially binds with the active site/protein binding site of the Spike, RdRP, and Mpro targets with a docking score of -8.22, -6.86, and -9.73 kcal/mole, respectively. Further, MS 3, 7-Hydroxyaloin B, 10-Hydroxyaloin A, showed -9.57, -7.07, -8.57 kcal/mole docking score against Spike, RdRP, and M^pro^ targets respectively. The MD simulation was performed to study the favorable confirmation and energetically stable complex formation ability of Isoquercetin and 10-Hydroxyaloin A phytochemicals in M^pro^-unbound/ligand bound/standard inhibitor bound system. The parameters such as RMSD, RMSF, Rg, SASA, Hydrogen-bond formation, energy landscape, principal component analysis showed that the lead phytochemicals form stable and energetically stabilized complex with the target protein. Further, MM-PBSA analysis was performed to compare the Gibbs free energy of the M^pro^-ligand bound and standard inhibitor bound complexes. The analysis revealed that the His-41, Cys145, Met49, and Leu27 amino acid residues were majorly responsible for the lower free energy of the complex. Drug likeness and physiochemical properties of the test compounds showed satisfactory results. Taken together, the study concludes that that the Isoquercetin and 10-Hydroxyaloin A phytochemical possess significant efficacy to bind SARS-Cov-2 M^pro^ active site. The study necessitates further *in vitro* and *in vivo* experimental validation of these lead phytochemicals to assess their anti-SARS-CoV-2 potential.

## Introduction

SARS-CoV-2 is a single-stranded RNA-enveloped virus whose gene fragments consist of structural and non-structural proteins. Some of the genes (*viz.*, E, M, N, and S) encode structural proteins, whereas some encode important non-structural proteins (*viz.*, papain-like protease- PLpro, 3-chymotrypsin-like/main protease-M^pro^, Spike-protein, and RNA-dependent RNA polymerase-RdRp) by the ORF region. The Spike-protein possesses two subunits (S1 and S2), which play important roles in the receptor recognition and membrane fusion process. The S1 subunit encompasses a receptor-binding domain (RBD) which interacts with the host receptor protein [such as angiotensin-converting enzyme 2 [ACE2] or TMPRSS2 protein], whereas the S2 subunit negotiates fusion of the virus to the host cellular membrane. These two events are critical to the entry of the viral particle into the host cells. After entry into the host cells, the viral RNA is released into the cell and the polyproteins are processed by the M^pro^ protein. The M^pro^ protease of SARS-CoV-2 is identified as a cysteine protease that possesses a catalytic dyad Cys145-His41 in the active site of the protease, responsible for its activity. M^pro^ plays an important role in the processing of replicase polyprotein and in the maturation of virus. The RNA-dependent RNA polymerase (RdRp) is essentially required for the viral RNA synthesis, which ultimately enhances the viral virulence. Thus, the SARS-CoV-2 Spike-protein, M^pro^, and RdRp proteins are important drugable target to mitigate the viral entry and virulence of the disease. Several preclinical and clinical studies on the chemically synthesized inhibitors of these drugable targets showed significant toxicity and other adverse effects. For instance, antiviral drugs such as lopinavir and remdesivir exert many side-effects in COIVD-19 patients (Barlow et al., 2020; Cao et al., 2020; Liu et al., 2020). Therefore, there is an urgent need to identify potential natural SARAS-CoV-2 drugable target inhibitors. Plant-based small molecules showed efficacy against different diseases/ailment including antiviral disease in *in silico* and *in vitro* experiments (Gupta et al., 2020; Kushwaha et al., 2020a; Kushwaha et al., 2020b; Kushwaha et al., 2021a). In the present study we selected two Indian medicinal plants (*Azadirachta indica* and *Aloe vera*) based on the rationale reviewed through the literature.

Verma (1974) first time reported antiviral potential (against potato virus X) in *Azadirachta indica* phytochemical (Verma, 1974). After that several antiviral activities have been reported in extract and/or pure isolated compounds from the plant against Coxsackie B group of viruses, Polio virus, Dengue virus type-2, HIV, Herpes simplex virus type 1, etc. Phytochemicals present in *A. indica* has potential to modulate the early event of viral replication cycle (Badam et al., 1999). SaiRam et al. (2000) proposed that neem phytochemicals have the ability to neutralize the viral particle before entering the cells (SaiRam et al., 2000). They also indicate the entry point inhibition potential in neem phytochemical against viral entry into the host cell. Parida et al. (2002) in their study also indicated that the neem phytoconstituents could inhibit the viral entry into the host cells (Parida et al., 2002). In a different study, Udeinya et al. (2004) reported that neem extract ameliorates adverse effects of human immunodeficiency virus (HIV) in human subjects possibly by inhibiting the cellular entry of the virus (Udeinya et al., 2004). In an interesting study, Xu et al. (2012) reported that the phytochemicals present in *A. indica* have the potential to neutralize the viral particle and inhibit the viral entry (Xu et al., 2012). Different studies reported the antiviral efficacy such as disruption of herpes simplex virus type 1 envelope, treatment of genital herpes virus, pigeon paramyxovirus type 1 Replication, anti-influenza activity, porcine epidemic diarrhea anti-viral activity of *Aloe vera* extracts/phytochemicals in various experimental models and clinical study (Sydiskis et al., 1991; Perfect et al., 2005; Dziewulska et al., 2018; Borges-Argáez et al., 2019). Interestingly, *A. vera* is an active ingredient of “Bioaron C” syrup, which is a herbal medicine known to prevent upper respiratory tract infections (URTIs). Besides, the plant has been used as medicine for URTIs for many decades (Glatthaar-Saalmüller et al., 2015). Over all the literature revealed the multimechanism-mediated antiviral potential in *A. Indica* and *A. vera* phytochemicals.

Our research group identified lead *A. Indica* phytochemicals having potential to bind human host protein TMPRSS2 involved in Spike-protein entry into the cell (Senapati et al., 2021). Matveeva et al. (2020) studied selected phytochemicals (n=1,911) from 55 plant species to find anti-SARAS-CoV-2 protein inhibitors. This showed about 36 phytochemicals per plant species. Similarly, few studies reported the main protease binding potential of only *A. Indica* phytochemicals, but they considered less number of phytochemicals (Garg et al., 2020; Adegbola et al., 2021; Umar et al., 2021). Lim et al. (2021) suggested *A. indica* as a potential herbal source of anti-SARS-CoV-2 agent with a multimodal efficacy of antiviral, anti-inflammatory, and immunomodulatory properties. Recently two different randomized controlled trials of *A. indica* extract/capsule on COVID-19 positive patients established the significant anti-SARS-CoV-2 potential in *A. indica* plant (Khan et al., 2020; Nesari et al., 2021). Similarly, few studies reported the *Aloe vera* phytochemicals as SARS-CoV-2 M^pro^ and RdRp binding potential (Mpiana et al., 2020; Abouelela et al., 2021; Balkrishna et al., 2021). These studies either considered fewer compounds or concerned only the *Aloe vera*–specific phytochemicals for the study. In the present study we targeted the three SARS-CoV-2 drugable targets (M^pro^, Spike-protein, and RdRp) and considered phytochemicals especially reported in *Aloe vera* plant. Keeping all the abovementioned facts, we selected the *A. indica* and *A. vera* phytochemicals (at larger scale) to revisit the identification of potential antiviral inhibitor of plant origin.

## Materials and Methods

### *Azadirachta indica* and *Aloe* *vera* (L.) Phytochemical Retrieval and Preparation

Compounds present in *Azadirachta indica* (n=93) and *Aloe vera* (L.) (n=37) were searched from different sources such as Science Direct, PubMed Central Google Scholar, Web of Science, PubMed, Scopus, Semantic Scholar, Medline, and Google Scholar (Kushwaha et al., 2021b). Marvin Sketch software (https://chemaxon.com/products/marvin) was used to prepare the structures of phytochemicals. Three-dimensional or two-dimensional structures of compounds were retrieved form NCBI PubChem database (Xie, 2010). Two-dimensional structures of compounds were converted into three-dimensional structure by using Open Babel software (O’Boyle et al., 2011). Using PyRx-Python prescription 0.8 for 200 steps, energy minimization of the ligands was performed by using Merck molecular force field (MMFF94) along with conjugate gradient optimization algorithm (Chitrala & Yeguvapalli, 2014).

### Protein Retrieval and Preparation

Crystal structures of target proteins, *viz.*, M^pro^ (PDB ID: 5RFS), Spike glycoprotein (PDB ID: 6VSB), RdRp (PDB ID: 6M71), were obtained from Protein Data Bank (https://www.rcsb.org/) with a resolution of 1.70, 3.46, and 2.90 Å, respectively. Three-dimensional structures of the targeted proteins were prepared for molecular docking using UCSF Chimera (Pettersen et al., 2004). All water molecules and ligands present in crystallized structure were removed. Steepest descent protocol with 100 steps and 0.02 step size along with conjugate gradient with 10 steps and step size 0.02 Å was applied for the energy minimization of the obtained protein structures.

### Molecular Docking

Auto Dock Tools 1.5.6 (ADT) was used to perform molecular docking of obtained proteins and ligands (Trott and Olson, 2010; Pushpendra et al., 2018). Proteins and ligands were loaded in ADT. Following this, the merging of non-polar hydrogens and torsions was used for the ligands by allowing the rotation of all rotatable bonds. Gestgeiger partial charge was assigned for the ligands. Docking calculations were performed for the all protein models. ADT tools were applied for the assignment of Kollman charges, polar hydrogen atoms, and solvation parameters. To explore the active binding region having differential efficacy, the Lamarckian Genetic Algorithm was used. The whole binding site was used to assign grid boxes of the target proteins to allow sufficient space for the ligands’ translational and rotational walk. Then 27,000 GA operations were generated with a single population of 150 individuals for every 30 independent runs. PyMOL software was used for visualization, and Discovery studio visualizer was used for the analysis of interface between receptors and ligands (DeLano, 2002).

### Molecular Dynamics Simulation

Following the docking studies, lead compounds obtained from both *Aloe vera* and *Azadirachta indica* were subjected to MD simulation studies for the evaluation of their binding efficacy and to illustrate the effect of lead compound binding on the internal dynamics of M^pro^ protein, along with standard inhibitor and unbound protein (Gupta et al., 2020; Singh et al., 2021). The GROMACS (Version 2020.4) was utilized to perform MD simulation (Abraham et al., 2015). GROMOS54a7 force field and single-point charge (SPC) water model was applied to conduct MD simulation of all complexes. Topologies and parameter files of the lead compounds and the standard inhibitor were generated using PRODRG server (Schüttelkopf and Van Aalten, 2004). All the complexes were simulated inside a cubic box with 1 Å of buffer distance. For the electro neutralization of the complexes, the respective number of ions were added. Bad contacts and clashes in the protein were resolved by energy minimization using 5,000 steps of steepest decent method. Following the energy minimization, all the complexes underwent two steps of equilibration, first 100 ps of NVT equilibration followed by 100 ps of NVT equilibration. In order to avoid the cold solute–hot solvent difficulty, temperature coupling was applied, which was achieved by indexing the system into non-water and water components by using *gmx make_ndx* module of GROMACS (Lemkul, 2019). The temperature of the system was maintained at 300°C by using Berendsen thermostat (Berendsen et al., 1984). Similarly, the pressure of the system was maintained by using Parrinello-Rahman barostat (Parrinello and Rahman, 1981). Long-range interaction present in the system was treated by applying the LINCS method (Hess et al., 1997). MD simulations were performed for 20,000 ps, and the coordinates were saved at every 1 ps for all the system. Structural and conformational analysis of all system was conducted using various analysis modules implemented in GROMACS package.

### Trajectory Analysis

Trajectories obtained following the molecular dynamics simulation were studied using tools available in the GROMACS package. The RMSD and RMSF of the lead phytochemical bound, standard inhibitor bound, and non-bound proteins were computed using *gmx rms* and *gmx rmsf* tools, respectively. The SASA and Rg were computed using *gmx sasa* and *gmx gyrate* tools, respectively. The energy calculations were performed using *gmx energy* tool. The change in the secondary structure of the unbound and ligand-bound test protein was analyzed using *gmx do_dssp* tool. The hydrogen bond formation was analyzed using *gmx hbond* tool. Visualization was performed using VMD and PyMol software (Humphrey et al., 1996; DeLano, 2002). Graphical representations were made using Grace Software (https://plasma-gate.weizmann.ac.il/Grace/).

### Principal Component Analysis

The PCA is a popular analytical tool to assess the reduction in the dimensionality of large datasets. It is also a widely applied technique in MD simulations for the illustration of the slow/functional movements in biomolecules (Gupta et al., 2020; Singh et al., 2021). The principal components for all three complexes were obtained by diagonalization and solvation of the eigenvalues and eigenvectors for the covariance matrices. The eigenvectors and eigenvalues demonstrate the direction and the magnitude of the motion, respectively. Calculation of covariance matrix was performed using GROMACS tool *gmx covar*. The *gmx covar* tool of the GROMACS package was applied to build and also diagonalize the covariance matrix. Further *gmx anaeig* was applied for the calculation of overlap between the computed principal components and coordinates of the trajectory.

### Free Energy Surface and Dynamical Cross-Correlation Matrix

Free energy surface (FES) is used to represent the possible conformation of the proteins in MD simulations. The change in possible conformation and the Gibbs free energy of the test protein (both in unbound and in ligand-bound complex) was studied. FES demonstrates the two variables that illustrate individual properties of the M^pro^-lead phytochemical bound, standard inhibitor bound, and unbound protein systems. The FES calculations also measured the conformational variability of the test systems. The FES calculation was made by calculating the probability distribution of the first two eigenvectors. For the DCCM analysis, GROMACS trajectory files (.xtc) were converted into Nanoscale Molecular Dynamics (NAMD) format (.dcd) (Phillips et al., 2020). Trajectory conversion was performed using VMD software. Bio3D package was used to calculate DCCM (Grant et al., 2006).

### g_mmpbsa Analysis

The *g_mmpbsa* is an important package used with GROMACS for the calculation of binding free energy (BE) of the ligand-bound complexes. The g_mmpbsa applies Molecular Mechanic/Poisson-Boltzmann Surface Area (MM-PBSA) approach for the BE calculation (Gupta et al., 2020). The BE was calculated for the known standard and the *A. indica* and *A. vera* lead phytochemicals bound at the active site of the target protein. The MD simulation trajectories for the last 5 ns (15–20 ns) were utilized to calculate the BE of the test complexes. The representation of the BE (ΔG_binding_) of the lead *A. indica* and *A. vera* phytochemical-bound protein complex was calculated using the following equation:

$$\Delta {\text{G}}_{\text{binding}}={\text{G}}_{\text{complex}}-({\text{G}}_{\text{protein}}+{\text{G}}_{\text{ligand}})$$

In above equation, G_complex_ demonstrates energy of the lead phytochemical/standard inhibitor bound test protein complex, and G_protein_ and G_ligand_ demonstrate the protein and ligand energy in water surrounded environment, respectively.

### ADMET Parameter and Bioactivity Prediction

Physicochemical properties and pharmacokinetics parameters of the identified compounds were evaluated by using the free web tool SwissADME (http://www.swissadme.ch/) (Daina et al., 2017). Boiled egg and bioavailability radar analysis was performed to evaluate the absorption and bioavailability of identified compounds.

## Results

**Figure 1:**
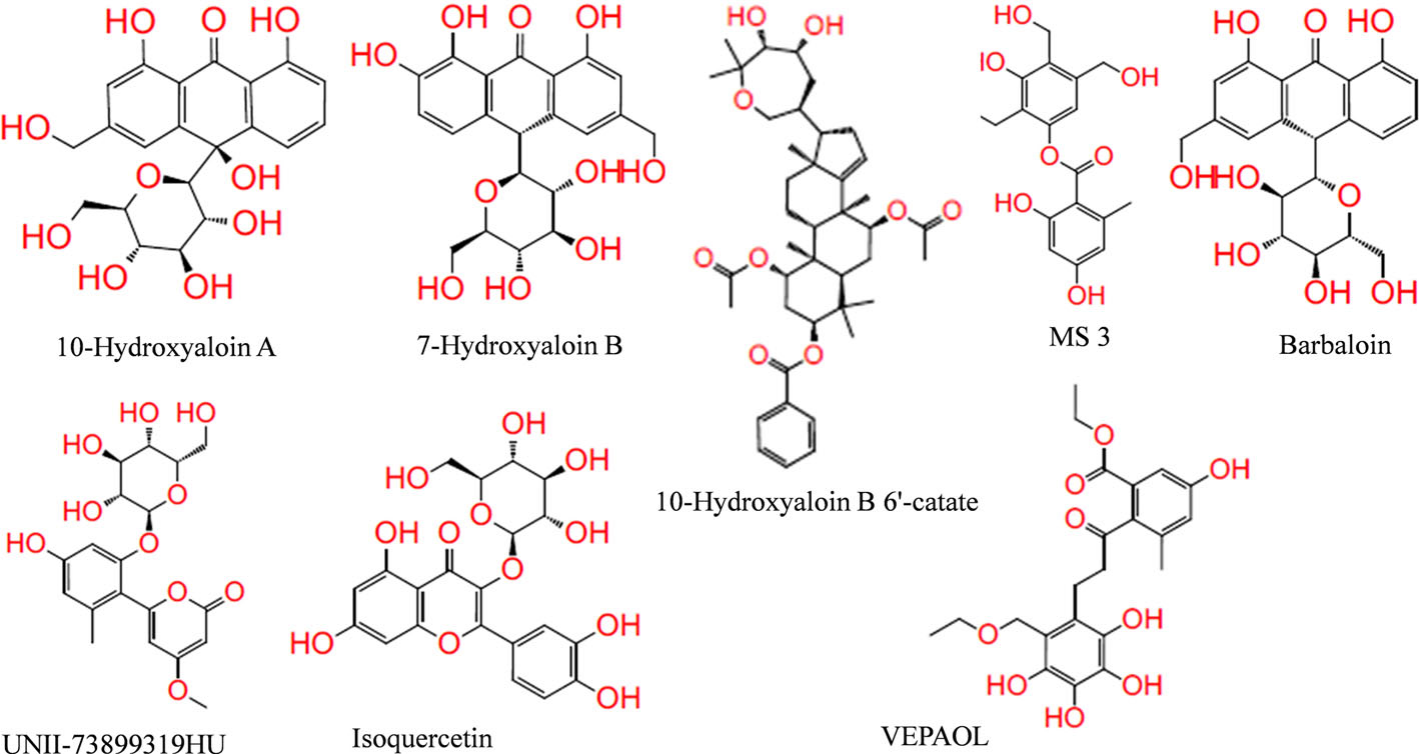
Structure of lead phytochemicals present in *Aloe vera* and *Azadirachta indica* having potential to bind at the active sites/protein binding sites of the SARS-CoV-2 drugable proteins.

In the present study, a total of 130 phytochemicals present in *Azadirachta indica* and *Aloe vera* were identified in literature-based search. The phytochemicals were docked at SARS-CoV-2 Spike, RdRp, and M^pro^ proteins. Docking score of the *Aloe vera* and *Azadirachta indica* lead phytochemicals (≤−6.0 kcal/mole cut-off value) against the test proteins is tabulated in **Tables 1**, **2**, respectively. The 2D structures of the top lead compounds against various test proteins are depicted in **Figure 1**.

**Table 1:** Docking score of *Aloe vera* phytochemicals against drugable targets of SARS-CoV-2 virus.

Protein	PubChem ID	Compound Name	Docking Score Kcal/mole
**M^pro^**			
	14889736	10-Hydroxyaloin A	-8.57
	158096	7-Hydroxyaloin B	-8.37
	10648253	10-Hydroxyaloin B 6’-catate	-8.35
	12305761	Barbaloin	-7.54
	6857486	CHEBI:35671	-7.45
	–	AA	-7.38
	160190	Aloesin	-6.97
	10207	Aloe-Emodin	-6.95
	100450	MS 3	-6.93
	5464178	1,3,6,8-Tetranitro-4,5-Dihydroxy-2-Hydroxymethylanthraquinones	-6.81
	5317657	UNII-73899319HU	-6.62
	5317653	CHEMBL518845	-6.6
	442866	Mycosporine	-6.41
**Spike Protein**			
	100450	MS 3	-9.75
	–	AA	-8.57
	12305761	Barbaloin	-8.03
	158096	7-Hydroxyaloin B	-7.86
	5904246	SCHEMBL4210152	-7.71
	14889736	10-Hydroxyaloin A	-7.46
	5317657	10-Hydroxyaloin A	-7.46
	6857486	CHEBI:35671	-7.25
	160190	Aloesin	-7.22
	11850	Galactitol	-7.1
	5317653	CHEMBL518845	-6.91
**RdRP**			
	158096	7-Hydroxyaloin B	-7.07
	6857486	CHEBI:35671	-6.96
	5317657	UNII-73899319HU	-6.77
	10648253	10-Hydroxyaloin B 6’-catate	-6.66
	14889736	10-Hydroxyaloin A	-6.59
	–	AA	-6.46

Docking Score with cutoff <−6.0 kcal/mole is considered. MS 3-3-hydroxy-4,5-bis(hydroxymethyl)-2-(3-methylbut-2-en-1-yl)phenyl 2,4-dihydroxy-6-methylbenzoate; AA-(10R)‐2,8‐dihydroxy‐6‐(hydroxymethyl)‐1‐methoxy‐10‐[(2R,3R,4R,5R,6R)‐3,4,5‐trihydroxy‐6‐(hydroxymethyl)oxan‐2‐yl]‐9,10‐dihydroanthracen‐9‐one.

**Table 2:** Docking score of *Azadirachta indica* phytochemicals against drugable targets of SARS-CoV-2 virus.

Protein	PubChem ID	Compound Name	Docking Score (Kcal/mole)
**M^pro^**			
	10813969	Isoquercetin	-9.73
	97343-95-8	VEPAOL	-9.43
	29803-85-8	Nimbidiol	-6.8
	11334829	Nimbidiol	-6.62
	21725519	Vilasinin	-6.55
	13875774	Nimbocinolide	-6.44
	5280863	Kaempferol	-6.35
	102090424	Melianin B	-6.33
	185704	Salannic (nimbidic) Acid	-6.27
	102146586	Azadirachtanin	-6.19
	23256847	Azadirachtol	-6.18
	6442906	Nimocinolide	-6.1
	105404-75-9	Margosinolide	-6.03
**Spike**			
	10813969	Isoquercetin	-8.22
	97343-95-8	VEPAOL	-7.71
	5280863	Kaempferol	-6.24
**RdRp**			
	10813969	Isoquercetin	-6.86
	184310	Isonimocinolide	-6.29

Docking Score with cutoff <−6.0 kcal/mole is considered.

**Figure 2:**
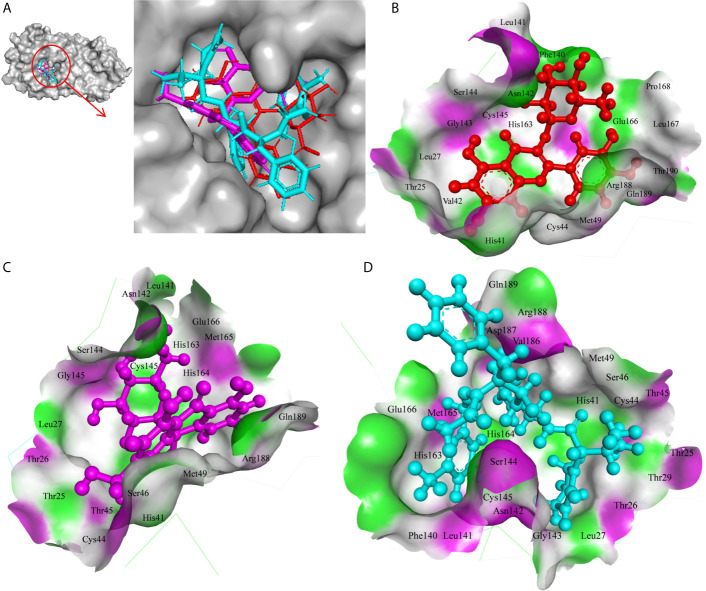
Interaction of lead phytochemicals and standard inhibitor at the M^pro^ active site. **(A)** Binding pose of lopinavir (Cyan), Isoquercetin (Red), and 10-Hydroxyaloin A (Pink). **(B)** Interaction of M^pro^ protein with isoquercetin shown with the hydrogen bond surface of receptor. **(C)** Interaction of M^pro^ protein with 10-Hydroxyaloin A shown with the hydrogen bond surface of receptor. **(D)** Interaction of M^pro^ protein with lopinavir shown with the hydrogen bond surface of receptor.

In the present study, Isoquercetin, MS 3, 7-Hydroxyaloin B, and 10-Hydroxyaloin A present in *Azadirachta indica* and *Aloe vera* medicinal plants showed potential binding against SARS-CoV-2 drugable targets, *viz.*, Spike-protein, RdRp, and Main protease proteins. The standard inhibitors lopinavir, VE607, and ribinavir were used to compare the docking efficacy of phytochemicals against M^pro^, Spike, and RdRp proteins, respectively. The 10-Hydroxyaloin A, 7-Hydroxyaloin B, and 10-Hydroxyaloin B 6’-catate *A. vera* compounds showed -8.57, -8.37, and -8.35 kcal/mole docking score against M^pro^ protein. Top three M^pro^ inhibitors present in *A. indica* (Isoquercetin, VEPAOL, and Nimbidiol) showed -9.73, -9.43, and -6.8 kcal/mole binding efficacy at the active site of the M^pro^ protein. The standard inhibitor lopinavir showed -5.33 kcal/mole binding efficacy. The binding pose of the lead M^pro^ inhibitors (Isoquercetin and 10-Hydroxyaloin A) and standard compound is depicted in **Figure 2A**. Amino acids involved in the binding with Isoquercetin, 10-Hydroxyaloin A, and standard inhibitor are depicted in **Figures 2B–D**. Asn142, residue of M^pro^, was involved in hydrogen bond formation with lopinavir. Besides, amino acid residues Ser46, Thr45, Cys44, Thr25, Thr24, Leu27, Gly143, Thr26, Met49, Arg188, His41, Asp187, Val186, His164, Met165, Ser144, Phe140, Leu141, Cys145, His163, Glu166, and Gln189 were involved in hydrophobic interaction with protease (**Table 3**). Isoquercetin formed hydrogen bonds with residues Glu166, Asn146, and His41. Gly143, Leu27, Thr25, Cys44, Val42, Met49, Cys145, Met165, Arg188, Gln189, Thr190, Pro168, Leu167, Leu141, Ser144, His163, and Phe140 amino acid residues of M^pro^ protein interacted with Isoquercetin by hydrophobic interaction (**Table 3**). 10-Hydroxyaloin A showed hydrogen bonding with His41, Leu141, Gly143, and Cys145 residues and furthermore interacted with Leu27, Arg188, Thr26, Glu166, Ser144, His164, Asn142, His163, Thr25, Thr45, Cys44, Ser46, Met49, Gln189, Met165 amino acid residues *via* hydrophobic interaction. The top three lead molecules from *Azadirachta indica* and *Aloe vera* (10-Hydroxyaloin A, 7-Hydroxyaloin B, 10-Hydroxyaloin B 6’-catate, Isoquercetin, VEPAOL, and Nimbidiol) showed H-bond interaction with His41, Leu141, Gly143, Cys145, Gln189, Glu166, Thr190, His164, Phe140, Asn146, Thr26, Asn142 residues. Moreover, the active phytochemicals showed hydrophobic interaction at the test protein active site (**Figure 2** and **Table 3**).

**Table 3:** Docking score and type of interaction of standard inhibitor and top three *Azadirachta indica* and *Aloe barbadensis* phytochemicals against SARS-CoV-2 M^pro^, spike, and RdRp proteins.

Target	PMID of ligand	Ligand name	Hydrogen-forming residues	Residues with hydrophobic interaction
**M^pro^**				
	**Standard**			
	92727	Lopinavir	**Asn142,** Ser46, Thr45, Cys44, Thr25, Thr24, Leu27, Gly143, Thr26, Met49, Arg188, His41, Asp187, Val186, His164, Met165, Ser144, Phe140, Leu141, Cys145, His163, Glu166, Gln189,	
	**Aloe**			
	14889736	10-Hydroxyaloin A	**His41, Leu141, Gly143, Cys145,** Leu27, Arg188, Thr26, Glu166, Ser144, His164, Asn142, His163, Thr25, Thr45, Cys44, Ser46, Met49, Gln189, Met165	
	158096	7-Hydroxyaloin B	**Leu141, His41, Gln189, Glu166, Thr190,** Met49, Asn142, His163, Pro168, Met165, Arg188,	
	10648253	10-Hydroxyaloin B 6’-catate	**Gly143, His164, Cys145, Phe140,** Ser46, Thr45, His41, Thr25, Cys44, Met49, Gln189, Met165, Leu141, Glu166, His163	
	**Neem**			
	10813969	Isoquercetin	**Glu166, Asn146, His41,** Gly143, Leu27, Thr25, Cys44, Val42, Met49, Cys145, Met165, Arg188, Gln189, Thr190, Pro168, Leu167, Leu141, Ser144, His163, Phe140	
	97343-95-8	VEPAOL	**Thr26, Asn142, Gly143,** Ser46, Gln189, Met49, His41, Leu27, His164, Met165, Glu166, His163, Ser144, Leu141, Phe140, HSI172, Cys145, Thr24, Thr25	
	29803-85-8	Nimbidiol	**Gly143, Thr26,** Met49, Gln189, Ser46, Asn142, Thr24, Thr25, Leu27, Ser144, Cys145, His164, His41, Met165, Glu166	
**Spike**				
	**Standard**			
	VE607	–	**Asp422,** Arg346, Thr345, Phe347, Arg509, Ser494, Lys417, Tyr421, Pro491, Leu492, Tyr351, Gln493, Ile418, Val350, Tyr495, Ile402, Ser349, Ala348, Phe497, Asp442	
	**Aloe**			
	100450	MS 3	**Ser494, Gln493,** Leu492, Pro491, Tyr421, Asn422, Lys417, Ile418, Tyr495, Val350, Gly496, Ser349	
	–	**BB**	**Phe497, Asp442, Phe347,** Tyr495, Ser349, Val401, Ser443	
	12305761	Barbaloin	**Tyr495, Ser494, Val401,** Gln493, Val350, Ser349	
	158096	7-Hydroxyaloin B	**Phe347, Asp442,** Arg346, Val401, Tyr495, Val350, Ser494, Ser349, Phe497	
	**Neem**			
	10813969	Isoquercitin	**Phe347, Val350, Asp442, Phe497,** Arg509, Arg346, Ala348, Val401, Ser349, Gln493, Tyr495, Tyr351, Ser494, Gln498, Pro499, Ser443	
	97343-95-8	Vepaol	**Val350, Asn422, Pro491, Tyr351,** Gln493, Leu492, Lys417, Ile418, Tyr495, Ser349	
**RdRp**				
	**Standard**			
	37542	Ribavirin	**Trp617, Asp618, Lys798, Glu811,** Tyr619, Ser814, Phe812, Asp761, Asp760, Ala762, Gly616, Trp800, Cys799,	
	**Aloe**			
	158096	7-Hydroxyaloin B	**Asp618, Tyr619, Lys621, Asp760, Lys798, Glu811,** Asp761, Asp623, Cys622, Pro620, Lys551, Arg553, Ser814	
	6857486	CHEBI:35671	**Asp760, Asp761, Ala762, His810, Glu811,** Asp618, Tyr619, Lys798, Cys799, Trp800, Gly616, Trp617, Val763, Phe812, Ser759, Ser814	
	5317657	UNII-73899319HU	**Arg553, Ser759, Asp760, Ser814, Lys798,** Lys545, Glu811, Phe812, Trp800, Cys799, Trp617, Tyr619, Trp617, Cys813, Asp618, Asp761, Leu758,	
	**Neem**			
	10813969	Isoquercetin	**Trp617, Asp618, Tyr619, Asp760, Lys798,** Pro620, Lys621, Cys622, Cys799, Trp800, Gly616, Ala762, Asp761, Glu811, Ser814, Ser759	
	184310	Isonimocinolide	**Tyr619, Lys621, Asp761, Ser814,** Asp618, Lys798, Asp623, Cys622, Pro620, Leu758, Asp760, Ser759, Cys813, Ala762, Phe812, Glu811, Trp617, Gly616, Trp800	

**BB**- (10R)‐2,8‐dihydroxy‐6‐(hydroxymethyl)‐1‐methoxy‐10‐[(2R,3R,4R,5R,6R)‐3,4,5‐trihydroxy‐6‐(hydroxymethyl)oxan‐2‐yl]‐9,10‐dihydroanthracen‐9‐one. The amino acids shown in bold were involved in hydrogen bond formation with their respective protein.

**Figure 3:**
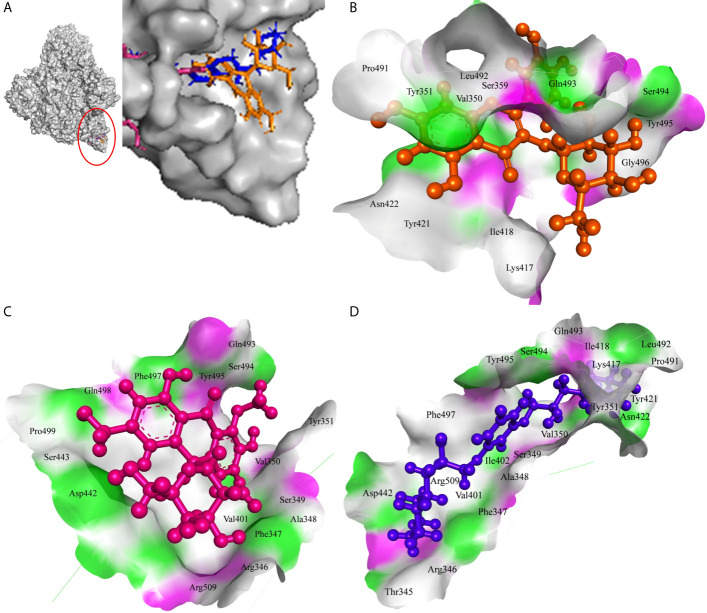
Interaction of lead phytochemicals and standard inhibitor with RdRp protein of SARS-CoV-2. **(A)** Binding pose of VE607 (Blue), Isoquercetin (Orange), and 10-Hydroxyaloin A (Pink) at the active site of the RdRp. **(B)** Interaction of RdRp active site with isoquercetin shown with the hydrogen bond surface of receptor. **(C)** Interaction of RdRp active site with 10-Hydroxyaloin A shown with the hydrogen bond surface of receptor. **(D)** Interaction of RdRp active site with VE607 shown with the hydrogen bond surface of receptor.

MS 3, **BB**, and Barbaloin phytochemicals in *A. vera* plant showed -9.57, -8.57, and -8.03 kcal/mole docking score against SARS-CoV-2 Spike-protein. The top three Spike-protein inhibitors present in *A. indica* (Isoquercetin, VEPAOL, and Kaempferol) showed -8.22, -7.71, and -6.24 kcal/mole binding efficacy at the protein binding site (S1) of the Spike protein. The standard inhibitor VE607 showed -7.81 kcal/mole binding efficacy. The binding pose of the lead Spike protein inhibitors (MS 3 and Isoquercetin) and standard compound is depicted in **Figure 3A**. Amino acids involved in the binding with MS 3, Isoquercetin, and standard inhibitor are depicted in **Figures 3B–D**. Asp422 residue of Spike protein S1 domain was involved in hydrogen bond formation with the VE607. Besides, several amino acid residues Arg346, Thr345, Phe347, Arg509, Ser494, Lys417, Tyr421, Pro491, Leu492, Tyr351, Gln493, Ile418, Val350, Tyr495, Ile402, Ser349, Ala348, Phe497, and Asp442 were involved in hydrophobic interaction with the protein-binding domain (**Table 3**). MS 3 formed hydrogen with residues Ser494, and Gln493. Leu492, Pro491, Tyr421, Asn422, Lys417, Ile418, Tyr495, Val350, Gly496, and Ser349 amino acid residues of the S1 domain of Spike protein interacted with MS 3 by hydrophobic interaction (**Table 3**). Isoquercetin showed hydrogen bonding with Phe347, Val350, Asp442, and Phe497. Furthermore, it interacted with Arg509, Arg346, Ala348, Val401, Ser349, Gln493, Tyr495, Tyr351, Ser494, Gln498, Pro499, Ser443 amino acid residues by hydrophobic interaction. The top two lead molecules from *Azadirachta indica* and top three *Aloe vera* (MS 3, **BB**; Barbaloin, Isoquercetin, and VEPAOL) showed H-bond interaction with Ser494, Gln493, Phe497, Asp442, Phe347 Tyr495, Val401, Val350, Val350, Asn422, Pro491, Tyr351 amino acid residues. Moreover, the active phytochemicals showed hydrophobic interaction at the test protein active site (**Figure 3** and **Table 3**).

**Figure 4:**
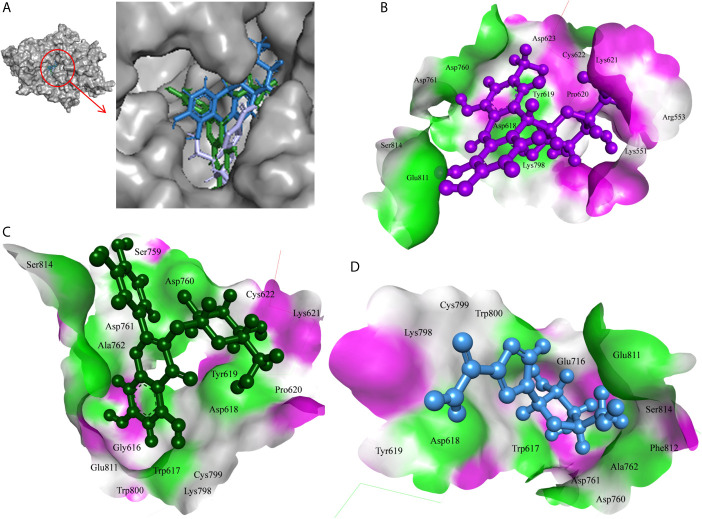
Interaction of lead phytochemicals and standard inhibitor with RdRp protein of SARS-CoV-2. **(A)** Binding pose of ribinavir (Cyan), Isoquercetin (Violet), and 10-Hydroxyaloin A (Green). **(B)** Interaction of Spike-protein RBD domain with isoquercetin shown with the hydrogen bond surface of receptor. **(C)** Interaction of Spike-protein RBD domain with 10-Hydroxyaloin A shown with the hydrogen bond surface of receptor. **(D)** Interaction of Spike-protein RBD domain with ribinavir shown with the hydrogen bond surface of receptor.

The 7-Hydroxyaloin B, CHEBI:35671, and UNII-73899319HU *A. vera* compounds showed -7.07, -6.96, and -6.77 kcal/mole docking score against SARS-CoV-2 RdRp protein. The top RdRp protein inhibitors (above the cut-off value) present in *A. indica* (Isoquercetin and Isonimocinolide) showed -6.86 and -6.29 kcal/mole binding efficacy at the active site of the protein. The standard inhibitor ribinavir showed -6.13 kcal/mole binding efficacy. The binding pose of the lead RdRp protein inhibitors (7-Hydroxyaloin B and Isoquercetin) and standard compound is depicted in **Figure 4A**. Amino acids involved in the binding with 7-Hydroxyaloin B, Isoquercetin, and standard inhibitor are depicted in **Figures 4B–D**. Trp617, Asp618, Lys798, and Glu811 residues of RdRp active site were involved in hydrogen bond formation with ribinavir. Besides, Tyr619, Ser814, Phe812, Asp761, Asp760, Ala762, Gly616, Trp800, and Cys799 amino acid residues showed hydrophobic interaction at the RdRp active site (**Table 3**). The 7-Hydroxyaloin B formed hydrogen with residues Asp618, Tyr619, Lys621, Asp760, Lys798, and Glu811. Furthermore 7-Hydroxyaloin B interacted with residues Asp761, Asp623, Cys622, Pro620, Lys551, Arg553, and Ser814 hydrophobically (**Table 3**). Isoquercetin formed hydrogen bonding with Trp617, Asp618, Tyr619, Asp760, and Lys798 amino acid residues. Furthermore, isoquercetin interacted with residues Pro620, Lys621, Cys622, Cys799, Trp800, Gly616, Ala762, Asp761, Glu811, Ser814, and Ser759 hydrophobically. The top two lead molecules from *Azadirachta indica* and top three from *Aloe vera* (7-Hydroxyaloin B, CHEBI:35671, and UNII-73899319HU, Isoquercetin, and Isonimocinolide) showed H-bond interaction with Asp618, Tyr619, Lys621, Asp760, Lys798, Glu811, Asp761, Ala762, His810, Arg553, Ser759, Ser814, and Trp617 amino acid residues of the RdRp protein. Moreover, the active phytochemicals showed hydrophobic interaction at the test protein active site (**Figure 4** and **Table 3**).

### Molecular Dynamics Simulation

**Figure 5:**
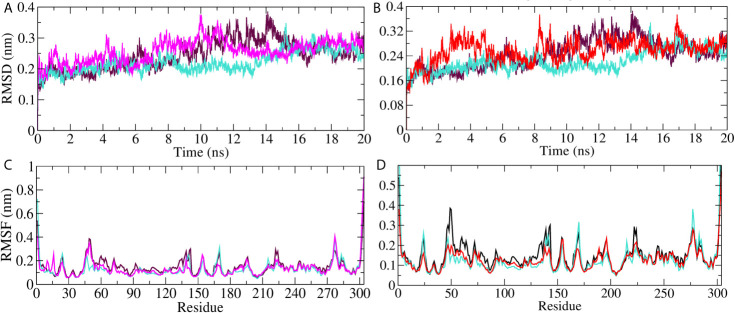
MD simulation trajectory plot of SARS-CoV-2 main protease in unbound and standard inhibitor ligand/natural lead compound bound complex. **(A)** The RMSD of the M^pro^ protein and lopinavir/10-Hydroxyaloin A lead compound complex during 20 ns MD simulation. **(B)** The RMSD of the M^pro^ and lopinavir/Isoquercetin lead compound complex during 20 ns MD simulation. **(C)** The RMSF values of M^pro^ protein and lopinavir/10-Hydroxyaloin A lead compound complex during 20 ns MD simulation. **(D)** The RMSF values of M^pro^ protein and lopinavir/Isoquercetin lead compound complex during 20 ns MD simulation Unbound protein, black color; *Aloe vera*–bound complex, pink; Neem-bound complex, red; and Lopinavir-bound complex is shown in light-blue color.

On the basis of docking results, we selected M^pro^ protein to perform molecular dynamics simulation study. The simulation was carried out on M^pro^ unbound, *A. indica*–, and *A. vera*–bound systems to study the dynamic behavior of the targeted protein. Simultaneously, the M^pro^ bound with its experimentally validated inhibitor was also performed to compare the results. Quality check parameters for the simulated system (temperature, pressure, potential/kinetic energy) were evaluated to check the validity of the performed simulations. Results showed that the quality check parameters were stable throughout the simulation period (**Supplementary Figures 1A–H**). MD simulation results for M^pro^-10-Hydroxyaloin A (MHA) complex, M^pro^-Isoquercetin (MIQ) complex, the unbound M^pro^ protein and M^pro^-Lopinavir (MLP) complex are shown in **Figures 5A, B**. The systems were stabilized and showed no significant alterations in density, temperature, volume, kinetic/potential/total energies, and pressure in unbound and lead ligand/standard inhibitor–bound protein/protein complex during the 20 ns MD simulation period (data not shown). Root mean square deviation (RMSD) of the MHA, MIQ, and MLP complexes did not show any significant deviation in comparison to unbound protein (**Figures 5A, B**). RMSD of 10-Hydroxyaloin A and Isoquercetin-bound M^pro^ complexes were comparable to known inhibitor-bound complexes. Root-mean-square fluctuation (RMSF) was assessed to analyze the impact of lead phytochemicals binding on the flexible portion of the targeted protein. Significant reduction in the RMSF values was observed in Isoquercetin-bound M^pro^ complex. The 10-Hydroxyaloin A and lopinavir-bound complexes did not show significant fluctuations in comparison to unbound protein (**Figures 5C, D**).

**Figure 6:**
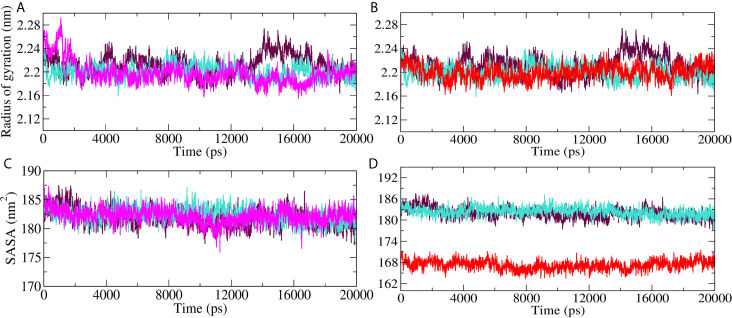
Radius of gyration (Rg) and solvent accessible surface (SASA) region of SARS-CoV-2 main protease in unbound and standard inhibitor ligand/natural lead compound–bound complex. **(A)** The Rg of the M^pro^ protein and lopinavir/10-Hydroxyaloin A lead–bound complex during 20 ns MD simulation. **(B)** The Rg of the solvated M^pro^ protein and lopinavir/Isoquercetin lead–bound complex during 20 ns MD simulation. **(C)** The SASA values of M^pro^ protein and lopinavir/10-Hydroxyaloin A lead–bound complex during 20 ns MD simulation. **(D)** The SASA values of M^pro^ protein and lopinavir/Isoquercetin lead–bound complex during 20 ns MD simulation. Unbound protein, black color; *Aloe vera*–bound complex, pink; Neem-bound complex, red; and Lopinavir-bound complex is shown in light-blue color.

Radius of gyration (Rg) and Solvent accessible surface area (SASA) analyses for M^pro^-10-Hydroxyaloin A (MHA) complex, M^pro^-Isoquercetin (MIQ) complex, M^pro^-Lopinavir (MLP) complex, and the unbound M^pro^ protein were compared, and the results are shown in **Figures 6A–D**. Result showed that 10-Hydroxyaloin A reduced the Rg value during the 20 ns simulation period in comparison to free protein and lopinavir-bound M^pro^ protein complex (**Figure 6A**). Isoquercetin showed similar Rg value pattern to lopinavir-bound M^pro^ complex (**Figure 6B**). Solvent accessible surface area analysis of the MD simulation trajectory revealed that 10-Hydroxyaloin A binding did not affect the SASA value significantly in comparison to MLP and pro systems (**Figure 6C**). It should be noted that the binding of Isoquercetin significantly reduced the SASA in comparison to lopinavir-bound and non-bound M^pro^ complexes during the entire 100 ns of MD simulation (**Figure 6D**).

**Figure 7:**
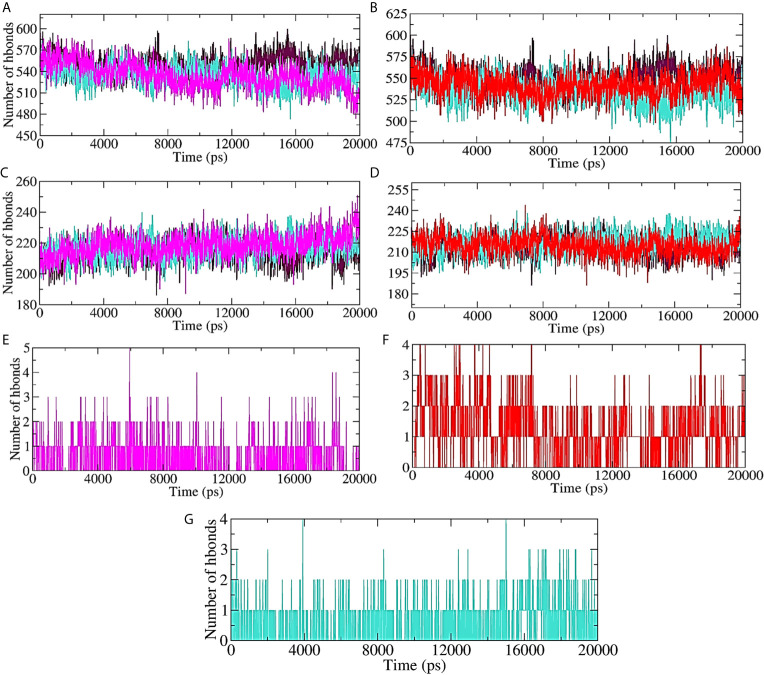
Plot of hydrogen bond formation during the MD simulation period of M^pro^ protein in ligand-bound and unbound conformation. Hydrogen bond formation between water and test protein in unbound, lopinavir-bound, **(A)** *Aloe vera* lead compound 10-Hydroxyaloin A–bound, and **(B)** Neem lead compound Isoquercetin-bound complexes. Intraprotein hydrogen bond formation in unbound, lopinavir–bound, **(C)** *Aloe vera* lead compound 10-Hydroxyaloin A–bound, and **(D)** Neem lead compound Isoquercetin-bound complexes. Hydrogen bond formation between M^pro^ complexed with **(E)** *Aloe vera* lead compound 10-Hydroxyaloin A, **(F)** Neem lead compound Isoquercetin, and **(G)** Lopinavir. Unbound protein, black color; *Aloe vera*–bound complex, pink; Neem-bound complex, red; and Lopinavir-bound complex is shown in light-blue color.

Hydrogen bond formation between the protein (ligand bound and unbound state) and the surrounding water molecules, within the protein (ligand bound and unbound state), as well as between the protein and lead compound/standard inhibitor was assessed, and the results are shown in **Figures 7A–G**. About 570 intermolecular hydrogen bonds were formed between unbound protein and water molecules. 10-Hydroxyaloin A binding with the M^pro^ protein significantly reduced the protein-water molecule H-bonding (**Figure 7A**). Isoquercetin showed lesser effect on H-bond formation between the test protein and surrounding water molecules (**Figure 7B**). The results were comparable with the standard inhibitor–bound protein complex (**Figures 7A, B**). Binding of 10-Hydroxyaloin A phytochemical to M^pro^ active site significantly increased (250) the intraprotein hydrogen bonding in comparison to unbound and lopinavir-bound M^pro^ protein (**Figure 7C**). Isoquercetin did not affect the interprotein H-bond formation in comparison to unbound and inhibitor-bound M^pro^ complex (**Figure 7D**). The H-bond formation between the protein and ligand (lead phytochemicals/standard inhibitor) showed that 10-Hydroxyaloin A and Isoquercetin binding favored H-bond formation in comparison to lopinavir binding at the active site of the M^pro^ protein during the MD simulation period (**Figures 7F, G**).

**Figure 8:**
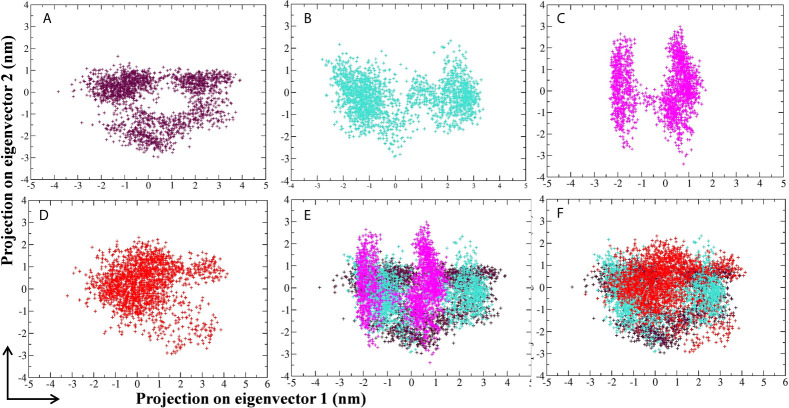
Projection of SARS-CoV-2 M^pro^ protein atoms in phase space along the first two principal eigenvectors. **(A)** Unbound M^pro^ protein. **(B)** Lopinavir-bound M^pro^ complex, **(C)** *Aloe vera* lead compound 10-Hydroxyaloin A–bound M^pro^ complex, **(D)** Neem lead compound Isoquercetin-bound M^pro^ complex, **(E)** Unbound M^pro^ and lopinavir/*Aloe vera* lead compound–bound complex, **(F)** Unbound M^pro^ and lopinavir/Neem lead compound–bound complex. Unbound protein, purple color; *Aloe vera*–bound complex, pink; Neem-bound complex, red; and Lopinavir-bound complex is shown in light-blue color.

Principal component analysis (PCA) of MD simulation trajectories was analyzed to show the collective motion of the active site–bound 10-Hydroxyaloin A and Isoquercetin-bound M^pro^ protein complexes. The results were compared with the unbound and standard inhibitor–bound M^pro^ protein/protein complex. The results of PCA analysis of the test system are depicted in **Figures 8A–F**. Results showed that binding of lead phytochemicals significantly decreased the collective motion of the M^pro^ protein in comparison to M^pro^ unbound and MLP systems.

**Figure 9:**
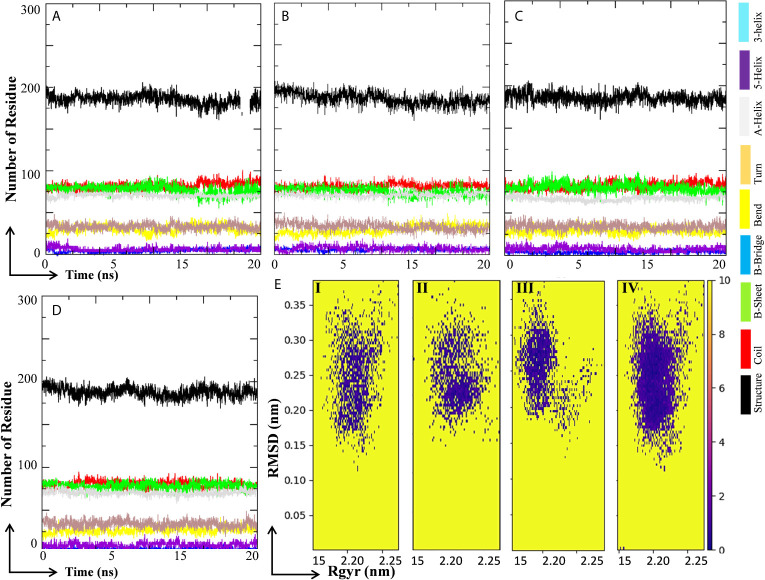
Secondary structure change and protein-ligand energy landscape of SARS-CoV-2 Main protease unbound and ligand-bound conformation. The secondary structure changes during the 20 ns MD simulation in **(A)** Unbound M^pro^ protein, **(B)** Lopinavir-bound M^pro^ complex, **(C)** *Aloe vera* lead compound 10-Hydroxyaloin A–bound M^pro^ complex, and **(D)** Neem lead compound Isoquercetin-bound M^pro^ complex. The free energy landscape of the **(Ei)** Unbound M^pro^ protein, **(Eii)** Lopinavir-bound M^pro^ complex, **(Eiii)** *Aloe vera* lead compound–bound M^pro^ complex, and **(Eiv)** Neem lead compound–bound M^pro^ complex.

Changes in secondary structure content (SSC) in the ligand-bound complex were studied for the change in structural behavior of the test protein in the presence of ligand at the active site. The SSC was studied in MHA, MLP, MIQ, and Mpro systems; and the results are depicted in **Figures 9A–D**. Results showed that the lopinavir binding did not affect significantly the various secondary structures in the Mpro protein (**Figures 9A, B**). A little fluctuation in the β-sheet of the M^pro^ protein was observed in 10-Hydroxyaloin A and Isoquercetin-bound M^pro^ protein complex (**Figures 9C, D**). To visualize the energy minima landscape of M^pro^-10-Hydroxyaloin A (MHA) complex, M^pro^-Isoquercetin (MIQ) complex, M^pro^-Lopinavir (MLP) complex, and the unbound M^pro^ protein, the FEL against PC1 (Rg) and PC2 (RMSD) was studied. The concise minimal energy area (blue color) obtained for the M^pro^-10-Hydroxyaloin A (MHA) and M^pro^-Isoquercetin (MIQ) complexes in comparison to lopinavir-bound and unbound protein is shown in **Figures 9Ei–iv**. The DCCM analysis was performed to find the effect of ligand binding on the correlated and anti-correlated motions of the target protein. It is evident from the DCCM analysis that the binding of 10-Hydroxyaloin A results in the significant decrease in both correlated (cyan color) and anti-correlated motions (pink color) of the M^pro^ protein in comparison to the unbound and lopinavir-bound M^pro^ protein (**Supplementary Figures 2A–C**). Binding of isoquercetin did not show significant effect on correlated and anti-correlated motions of target protein in comparison to the unbound and lopinavir-bound protein (**Supplementary Figure 2D**).

**Figure 10:**
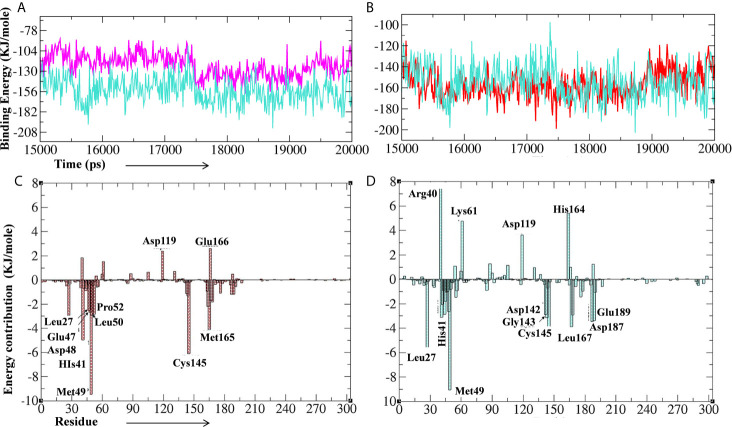
Binding energy of the lead phytochemicals and contribution energy of amino acid residues in the g_mmpbsa analysis. **(A)** Binding energy plot of M^pro^-lopinavir (light-blue color) and 10-Hydroxyaloin A (pink color) complex. **(B)** Binding energy plot of M^pro^-lopinavir (light-blue color) and Isoquercetin (red color) complex. **(C)** Residue contribution plot of M^pro^-10-Hydroxyaloin A complex. **(D)** Residue contribution plot of M^pro^- Isoquercetin complex.

Further, we performed the MM-PBSA analysis of the last 5 ns (15–20 ns) of the lead phytochemical/standard inhibitor–bound M^pro^ complex trajectories (obtained from 20 ns MD simulations) to calculate the thermodynamics parameters of the complex such as binding free/van der Waals/electrostatic/polar solvation energies (ΔE_binding_; E_vdw_; E_elec_; ΔE_polar_ respectively) and SASA (**Figures 10A–D** and **Table 4**). The binding energy of *A. indica* and *A. vera* lead phytochemical–bound M^pro^ complexes was stable during the analysis of the simulation trajectory (**Figures 10A, B**). The MM-PBSA data analysis also allows us to calculate the contribution of amino acid residues in the studied parameters (total binding energy). The total binding free energy was decomposed into the per amino acid residue contribution energy. The results for the amino acid residue contribution in the binding of lead phytochemicals are shown in **Figures 10B, C**. The M^pro^-10-Hydroxyaloin A binding analysis showed that the amino acid residues Met49, His41, Asp48, Cys145, Met165, Pro52, Leu50, and Leu27 played significant roles in the complex formation (**Figure 10A**). Similarly, M^pro^-Isoquercetin binding involved the significant contribution of leu27, His41, Met49, Asp142, Gly143, Leu167, Asp187, and Glu189 amino acid residues (**Figure 10B**).

**Table 4:** Binding free energy for Main protease complexes with standard inhibitor and lead phytochemical–bound complexes.

BE type	BE values MLP	BE values MHA	BE values MIQ
Δ*E*_binding_ (kj/mol)	-154.850 +/− 16.491	-122.513 +/− 14.118	-156.650 +/− 12.970
SASA (kj/mol)	-25.582 +/− 1.701	-15.250 +/− 1.268	-19.524 +/− 1.316
Δ*E*_polar solvation_ (kj/mol)	145.474 +/− 18.030	49.261+/− 8.881	71.794 +/− 13.762
Δ*E*_Electrostatic_ (kj/mol)	-29.830 +/− 9.759	-7.357+/− 7.080	-5.438+/− 7.347
Δ*E*_Van der Waal_ (kj/mol)	-244.912 +/− 16.819	-149.166+/− 12.217	-203.481 +/− 14.122

BE, Binding Energy; MLP, M^pro^-Lopinavir complex; MHA, M^pro^-10-Hydroxyaloin A complex; MIQ, M^pro^- Isoquercetin complex.

**Figure 11:**
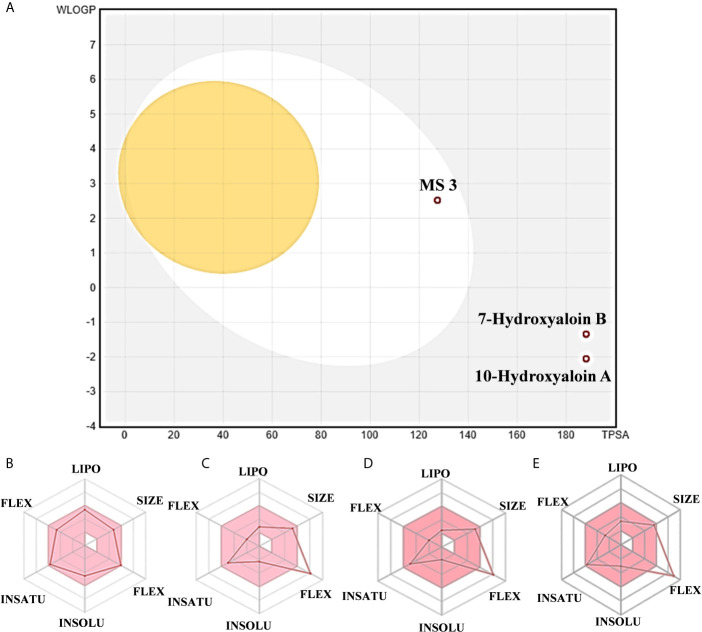
Boiled egg diagram and bioavailability radar map of lead phytochemicals. **(A)** Boiled egg model of MS 3, 10-Hydroxyaloin A, and 7-Hydroxyaloin B phytochemicals. The isoquercetin molecule value was out of range of the boiled egg plot. Bioavailability radar map of **(B)** MS 3, **(C)** 10-Hydroxyaloin A, **(D)** 7-Hydroxyaloin B, and **(E)** Isoquercetin. LIPO, lipophilicity; SIZE, molecular weight; POLAR, polarity; INSOLU, insolubility; INSATU, insaturation; FLEX, rotatable bond flexibility.

The boiled egg diagram for the lead phytochemicals (MS 3, 10-Hydroxyaloin A, 7-Hydroxyaloin B, and Isoquercetin) was prepared to study their blood–brain barrier crossing potential. All the phytochemicals showed satisfactory results (**Figure 11A**). The bioavailability radars for the lead phytochemicals against the Spike-protein, M^pro^, and RdRp drugable targets of SARS-CoV-2 were studied, and the results are shown in **Figure 11**. The radar plot shows the important drug-likeness properties such as lipophilicity, molecular weight, polarity, insolubility, insaturation, and rotatable bond flexibility of the test compounds. The bioavailability radars of the lead compounds were in the range and satisfactory. The 10-Hydroxyaloin A, 7-Hydroxyaloin B, and Isoquercetin showed little deviation from the required flexibility region of the radar (**Figures 11C–E**). Further, the various physiochemical properties of MS 3, 10-Hydroxyaloin A, 7-Hydroxyaloin B, and Isoquercetin are summarized in **Table 5**.

**Table 5:** Physiochemical properties of the lead phytochemicals.

Molecule	MS 3	7-Hydroxyaloin B	10-Hydroxyaloin A	Isoquercetin
**Formula**	C_21_H_24_O_7_	C_21_H_22_O_10_	C_21_H_22_O_10_	C_21_H_20_O_12_
**MW(g/mol)**	388.41	434.4	434.39	464.38
**HA**	28	31	31	33
**AHA**	12	12	12	16
**FCsp3**	0.29	0.38	0.38	0.29
**HBA**	7	10	10	12
**HBD**	5	8	8	8
**MR**	104.85	103.98	103.01	110.16
**TPSA**	127.45	188.14	188.14	210.51
**ESOL-S (mg/ml)**	1.08	2.08	6	4.23
**ESOL-C**	MS	S	VS	S

MW, Molecular weight; HA, Heavy atoms; AHA, Aromatic heavy atoms; FCsp3, Fraction Csp3; HBA, Hydrogen bond acceptor; HBD, Hydrogen bond donors; MR, Molar refractivity; TPSA, The polar surface area; ESOL-S, ESOL-Solubility; ESOL-C, ESOL Class; S, Soluble; PS, Poorly soluble; MS, Moderately soluble; GIA, GI absorption; BBB-P, BBB permeant; Pgp-S, Pgp substrate; BS, Bioavailability score.

## Discussion

In the present study we found the potential interaction of phytochemicals with the drugable targets of SARS-CoV-2-mediated infection in human. For this we targeted the three key steps of the viral pathogenesis, *viz.*, viral entry into the host cell (Spike-protein), conversion of viral precursor to functional proteins (M^pro^), and viral genome replication (RdRp). Interaction of Spike-protein RBD with the ACE-2 receptor (human host protein) is a critical step in viral entry into the host cell (Senapati et al., 2020). Recently Shang et al. (2020) reported that residues 455, 482–486, 493, 494, and 501 are critical SARS-CoV-2 S1 domain amino acids involved in interaction with human ACE-2 protein (Shang et al., 2020). Our research group reported inhibitory potential of phytochemicals against SARS-CoV-2 drugable proteins. The *in silico* inhibitory potential of *Curcuma longa* and *Withania somnifera* phytochemicals against M^pro^ protein of SARS-CoV-2 has been reported recently (Gupta et al., 2020; Kushwaha et al., 2021c). The identified lead phytochemicals showed significantly increased binding potential at the M^pro^ active site in comparison to standard inhibitors in molecular docking and molecular dynamics simulation study (Gupta et al., 2020; Kushwaha et al., 2021c). In the present study, we found that lead *A. indica* and *A. vera* phytochemicals interacted with two of the critical amino acids (Gln493 and Ser494) (**Table 3**). Isoquercetin formed hydrogen bonding, while MS 3 showed hydrophobic interaction with the Gln493 and Ser494 amino acids, which are critical for viral Spike-protein and human host ACE-2 binding receptor. MS 3 showed H-bonding with the Asn422 amino acid residue, which is reported to be involved in ACE-2 binding of Spike-protein (Gordon et al., 2020). Lead phytochemicals (binding efficacy below <−6.0 kcal/mole) also showed hydrophobic and hydrogen bond interaction with the key amino acids involved in Spike-protein RBD domain and ACE-2 protein-protein interaction (Asp442, Ser494, and Gln493). The results indicate that Isoquercetin and MS 3 have potential to disrupt spike glycoprotein-ACE-2 protein-protein interaction by binding at SARS-CoV-2 RBD. Thus, the lead molecules might inhibit viral entry into the cell. It has been shown that RdRp inhibitors (such as remdesivir) potentially block the RNA synthesis and thereby delay the chain termination process in SARS-CoV-2 RNA synthesis (Gordon et al., 2020). SARS-CoV-2 RdRp inhibitors are being studied in clinical trials in various countries of the world. Binding of *A. indica* and *A. vera* lead compounds at RdRp active site showed interaction with similar amino acids (Asp618, Tyr619, Asp760, Lys798, Glu811, and Asp761), indicating the SARS-CoV-2 RdRp inhibition potential in the test medicinal plants (**Table 3**). The SARS-CoV-2 RNA–mediated translation generated polyproteins that later on cleaved at some specific sites by Main protease enzyme to produce functional proteins. This step aids in the virulence of SARS-CoV-2. Literature reports that a Cystine-Histidine dyad is essential for the protease activity of M^pro^. Besides, the alanine, glycine, glutamate, serine, and leucine residue also play important roles in the cleavage catalysis process (Gupta et al., 2020). In the present study, the 10-Hydroxyaloin A and Isoquercetin showed hydrogen bond interaction with the Cys and His residues, indicating the Cys-His dyad disruption potential of the lead phytochemicals. The dyad disruption might lead to the decreased M^Pro^ activity, which in turn inhibits the production of functional protein. Thus, the 10-Hydroxyaloin A and Isoquercetin might be involved in the SARS-CoV-2 virulence mitigation.

RMSD is a parameter that computes the distance between protein atoms. The average distance between the atoms in unbound and ligand/standard inhibitor–bound targeted protein allows us to assess the comparative conformation and stability of the protein (Gupta et al., 2020). The present study indicates that the binding of 10-Hydroxyaloin A and Isoquercetin did not affect the conformational stability of the M^Pro^ protein (**Figures 5A, B**). The result indicates the stable M^pro^-10-Hydroxyaloin A (MHA) and M^pro^-Isoquercetin (MNB) complex formation. RMSF is an important parameter to assess the fluctuation of protein atoms across the time duration from a reference position. This allows us to study the comparative fluctuations in the portion of target protein (residue) before and after the ligand binding. In this study, binding of 10-Hydroxyaloin A and Isoquercetin showed the stabilization of the 40–45 and 140–165 amino acid residues during the simulation time (**Figures 5C, D**). The key amino acids required for the catalytic activity of the test protein fall in these areas. Thus, it might be inferred that the lead phytochemicals tightly bind with the key amino acids and stabilize the active site of the protein. In the previous studies in our laboratory, we found that phytochemical binding mediated lesser fluctuations in target protein amino acid residues (Gupta et al., 2020; Kushwaha et al., 2021c). Radius of gyration (Rg) provides valuable information about the folding of regular secondary structure of the targeted protein into the tertiary or functional structure before and after binding of the ligand/inhibitor molecule. In comparison to unbound protein, the 10-Hydroxyaloin A and Isoquercetin binding mediated decreased Rg value during the simulation period, indicating the compact and stabilized folding in the ligand-bound complexes (**Figures 6A, B**). The binding of a ligand at the active site of a protein surrounding a solvent is a solvent-substitution process. Thus, calculations of Solvent accessibility surface area (SASA) of ligand-bound and unbound protein give important information about the potential ligand binding. Isoquercetin binding mediated significantly decreased SASA value of the complex (in comparison to standard inhibitor–bound and non-bound protein), indicating the stabilized protein structure/active site throughout the simulation period (**Figures 6C, D**). Although the various types of interactions among ligand and targeted protein are involved in the complex stabilization process, hydrogen bond formation plays a significant role in this process. The greater the number of hydrogen bond incidence during the ligand-protein complex formation, the greater the stability of the complex. In the present study, the increased hydrogen formation potential (in comparison to standard inhibitor) of the 10-Hydroxyaloin A and Isoquercetin phytochemicals during the simulation period indicates the potential stability of the protein complex (**Figures 7E–G**). The greater number of hydrogen bond formation in the test compound interaction with the M^pro^ protein corroborates the more negative docking score of the lead compounds in comparison to standard inhibitor. The PCA and free energy landscape results (compact structure and increased centric energy respectively) indicate the compactness of the M^pro^ protein structure after binding the lead molecules at the active site of the protein (**Figures 8**, **9**). The analysis of the various secondary structures of the test protein in the presence of 10-Hydroxyaloin A and Isoquercetin indicated the involvement of β-sheet and 5’-helix in the interaction (**Figure 9**). The DCCM analysis indicated that the 10-Hydroxyaloin A inhibited the amino acid motion in the test protein, but isoquercetin revealed similar pattern of amino acid motions in standard inhibitor–bound protein. Over all, the analysis of the MD simulation trajectory revealed the stable and energetically favorable complex formation in the presence of lead phytochemicals. The MM-PBSA analysis showed the contribution of amino acids in the ligand-protein binding (**Figure 10**). It should be noted that the His41 and Cys145 amino acid residues involved in catalytic dyad were contributing to the interaction of lead phytochemicals and the M^pro^ protein. The results substantiate the potential binding and active site inhibition potential in the lead molecules. Over all, the boiled egg diagram, the bioavailability radar, and the physiochemical properties of the *A. indica* and *A. vera* lead phytochemicals showed the drug-like potential that could be utilized for anti-SARS-CoV-2 drug discovery (**Figure 11**).

## Conclusion

Plant-derived compounds possess single and/or multitargeted therapeutic potential against various diseases including viral disease. Thus, the computer-based identification of phytochemicals present in the medicinal plants is the need of time to identify potential inhibitors of SARS-CoV-2 drugable targets. The natural compounds possess less toxicity and associated side-effects, which make them a suitable candidate for drug discovery. In the present molecular docking study, we found that the phytochemicals present in *A. vera* and *A. indica* medicinal plants possess significant binding potential at the active site/protein-protein interaction sites of the SARS-COV-2 drugable targets (Spike-protein, RdRp, and M^pro^ proteins). Further, the molecular dynamic (MD) simulation and MMPBSA calculations revealed that 10-Hydroxyaloin A and Isoquercetin phytochemicals present in the *A. vera* and *A. indica*, respectively, stabilize the structure and energy of the M^pro^-ligand complex. More importantly, the lead phytochemicals showed disruption of His-Cys dyad at the active site of the M^pro^ protein required for its catalytic activity. In MMPBSA analysis, we found that the His and Cys residues contributed significantly in the binding free energy of the complex. Overall, we conclude that the *A. vera* and *A. indica* Indian medicinal plants could be taken as source of lead anti-SARS-CoV-2 agents for future drug discovery.

## Data Availability Statement

The original contributions presented in the study are included in the article/**Supplementary Material**. Further inquiries can be directed to the corresponding author.

## Author Contributions

SK designed and conceptualized the study. PK, AS, TB, and AY contributed in the data generation. SK wrote the manuscript. The figures and tables were developed by KP, MS, and AS. All authors contributed to the article and approved the submitted version.

## Conflict of Interest

The authors declare that the research was conducted in the absence of any commercial or financial relationships that could be construed as a potential conflict of interest.

## Publisher’s Note

All claims expressed in this article are solely those of the authors and do not necessarily represent those of their affiliated organizations, or those of the publisher, the editors and the reviewers. Any product that may be evaluated in this article, or claim that may be made by its manufacturer, is not guaranteed or endorsed by the publisher.
