# Supplementary material for: Identification of Natural Inhibitors Against SARS-CoV-2 Drugable Targets Using Molecular Docking, Molecular Dynamics Simulation, and MM-PBSA Approach
Source: Front Cell Infect Microbiol. 2021 Aug 12;11:730288. doi: 10.3389/fcimb.2021.730288 (PMC8387699; doi:10.3389/fcimb.2021.730288)
Supplement: Supplementary file 1 [file DataSheet_1.docx]

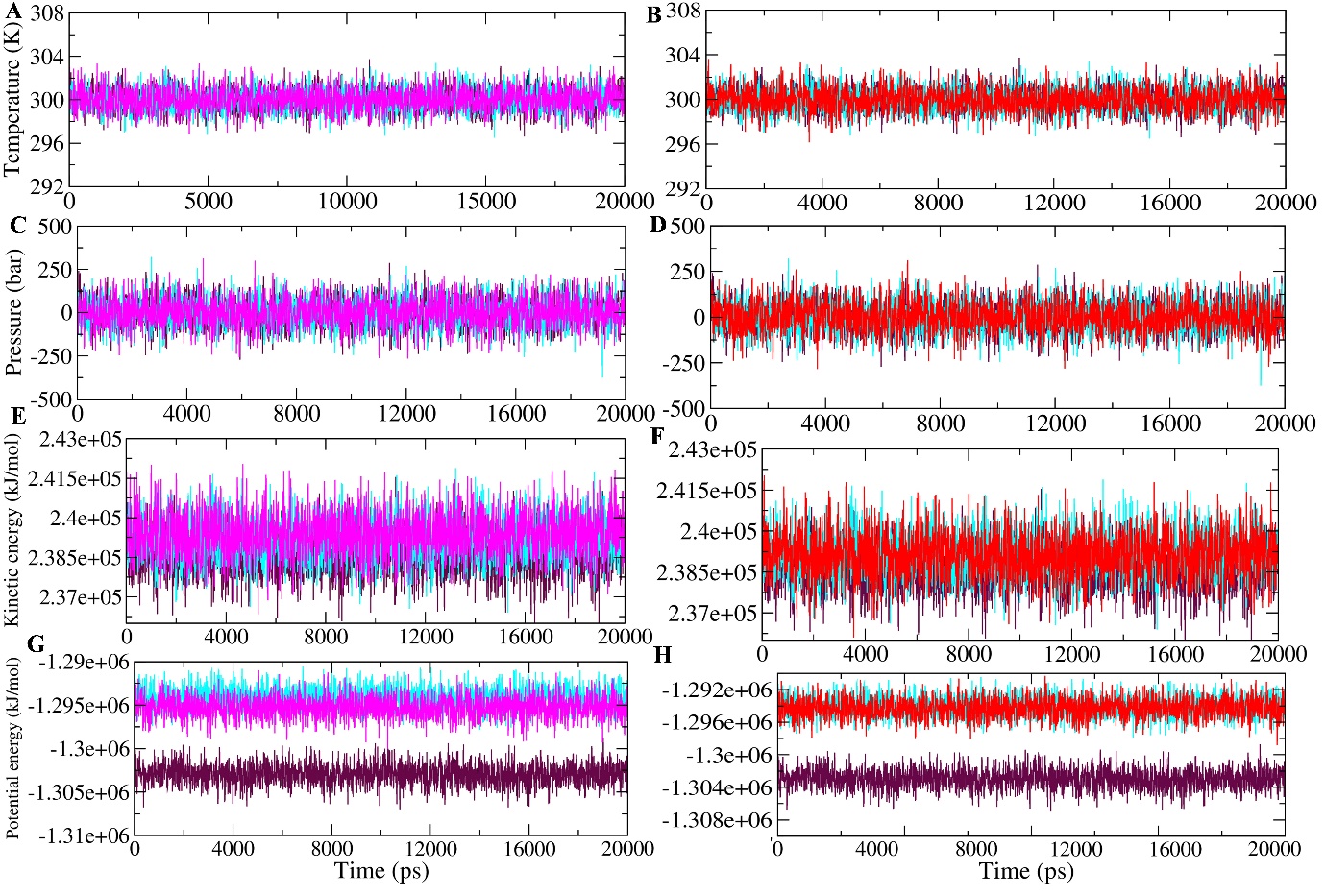


**Supplementary Figure 1: Quality check related parameters of the simulated system.** **(A)** Temperature of the Mpro protein, and lopinavir/10-Hydroxyaloin A lead compound complex during 20 ns MD simulation **(B)** Temperature of the Mpro and lopinavir/Isoquercetin lead compound complex during 20 ns MD simulation. **(C)** Pressure of the Mpro protein, and lopinavir/10-Hydroxyaloin A lead compound complex during 20 ns MD simulation **(D)** Pressure of the Mpro and lopinavir/Isoquercetin lead compound complex during 20 ns MD simulation. **(E)** Potential energy of the Mpro protein, and lopinavir/10-Hydroxyaloin A lead compound complex during 20 ns MD simulation. **(F)** Potential energy of the Mpro and lopinavir/Isoquercetin lead compound complex during 20 ns MD simulation. **(G)** Kinetic energy of the Mpro protein, and lopinavir/10-Hydroxyaloin A lead compound complex during 20 ns MD simulation. **(H)** Kinetic energy of the Mpro and lopinavir/Isoquercetin lead compound complex during 20 ns MD simulation. Unbound protein-maroon color; Alovera bound complex-Pink; Neem bound complex-Red; and Lopinavir bound complex is shown in cyan.


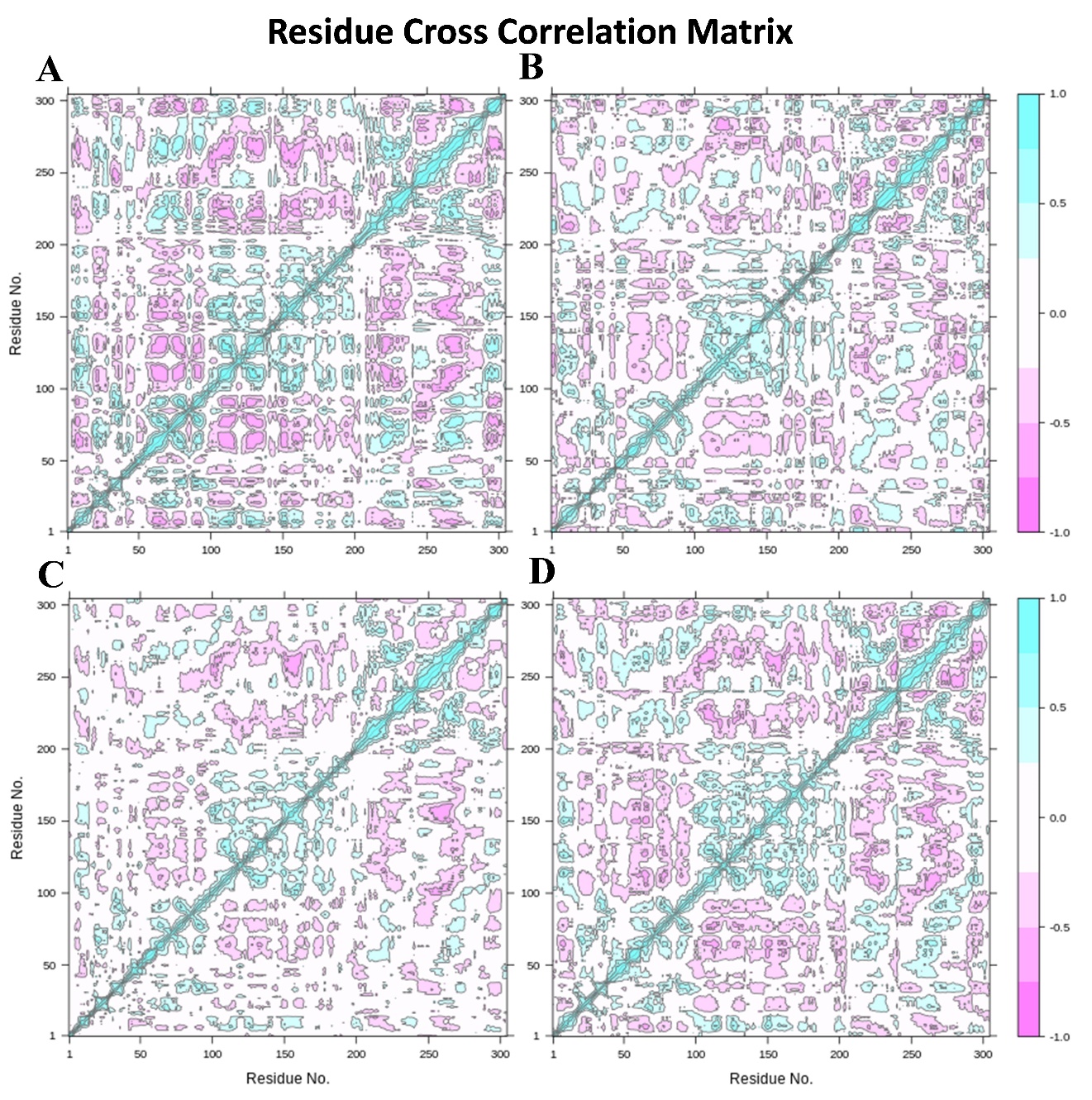


**Supplementary Figure 2: Dynamical cross-correlation matrix (DCCM) analysis of simulated systems.** **(A)** DCCM analysis of unbound Mpro protein. **(B)** DCCM analysis of Mpro protein bound with lopinavir. **(C)** DCCM analysis of Mpro protein bound with 10-Hydroxyaloin A. **(D)** DCCM analysis of Mpro protein bound with isoquercitin.
